# Prospective study on factors affecting the prognosis of oral cancer in a Chinese population

**DOI:** 10.18632/oncotarget.13842

**Published:** 2016-12-09

**Authors:** Fengqiong Liu, Fa Chen, Jiangfeng Huang, Lingjun Yan, Fangping Liu, Junfeng Wu, Yu Qiu, Xiaoyan Zheng, Rongzhao Zhang, Lisong Lin, Baochang He

**Affiliations:** ^1^ Department of Epidemiology and Health Statistic, Fujian Provincial Key Laboratory of Environment factors and Cancer, School of Public Health, Fujian Medical University, Fujian, China; ^2^ Department of Stomatology, The First Affiliated Hospital of Fujian Medical University, Fujian, China

**Keywords:** oral cancer, prognosis, BMI, chemotherapy, radiotherapy

## Abstract

This study was performed to identify the factors affecting prognosis of oral cancer patients. 1240 pathologically confirmed oral cancer patients were included. The sociodemographic and clinical characteristics of all patients were collected. Univariate and multivariate Cox proportional hazards models were used to assess potential prognostic factors for survival. 1240 oral cancer patients were followed up for 49235.00 person months, and the 5-year overall survival rate was 64.38%. Both univariate and multivariate Cox regression analysis indicated that Body Mass Index < 18.5 kg/m^2^ (vs 18.5–23.9 kg/m^2^), age ≥ 55 years (vs < 55 years), clinical stages of II-IV (vs stage I), and poor differentiation (vs well differentiation) were associated with worse survival of oral cancer patients. While surgery (vs non-surgery) and origin of urban area (vs rural area) were protective factors. However, no significant association was found between adjuvant therapy and survival in oral cancer patients.

## INTRODUCTION

Oral cancer is the most common cancer among head and neck carcinomas, which is becoming a major health problem particularly in developing countries [1]. According to recent data from the National Central Cancer Registry of China, the age-standardized rates of oral cancer in the Chinese population were 2.22/100,000 in 2011 and data shows that there is an increasing tendency in the incidence [2]. Moreover, the prognosis of oral cancer was not significantly improved in the past decades, a relative overall survival rate of 81.7, 61.7 and 54.9% at 1, 3 and 5 years, respectively in the US [3].

Despite being heavily influenced by age, tumor stage, sites, and histological grading, the survival rate of oral cancer patients is influenced by many other factors such as the time between disease and perception, related treatment, access to health-care services, educational levels and occupation of the patients, behavioral/cultural factors, exposure to risk factors such as chewing tobacco [4–8].

Although several studies evaluated the influence of these social factors on survival rate of oral cancer, most of the studies do not make proper assessments considering the potential confounding factors or with limited sample size. Thus far, large scale prospective study about prognosis of oral cancer in China was still absent. The objective of this study is to determine whether clinical features, histopathology, and socio-economic status will influence the survival of patients with oral cancer in Southeast China.

## RESULTS

### The clinicopathological characteristics and overall survive rate of oral cancer patients

795 males and 445 females (1.78:1) were included in the analysis with a median age of 57 years old. As shown in Table 1, there were 464 (43.9%) early stage (I–II) and 593(56.1%) advanced (III–IV) oral cancer patients. Well differentiated tumors were observed in 472 (46.5%) patients, moderately differentiated tumors in 410 (40.1%) patients, poorly differentiated tumors in 134 (13.2%) patients. For all the 1240 patients, 144 patients (11.6%) did not receive surgery. Among the 1096 patients who undertook surgery, 390 patients (35.6%) received surgery alone, 236 patients (21.5%) received surgery and chemotherapy (CT), 147 patients (13.4%) received surgery and radiotherapy (RT), and 323 patients (29.5%) received surgery and chemoradiotherapy (CRT).

**Table 1:** Results of univariate analysis of prognostic factors for survival in oral cancer

Characteristic	Number of Censored (%)	Number of death (%)	5-year survival rate (%)	log-rank *P*	*HR* (95%CI)
Gender					
male	520(60.82)	275(71.43)	61.78	< 0.001	1.00
female	335(39.18)	110(28.57)	69.18		0.67 (0.54,0.84)
Age					
< 55	379(44.33)	136(35.32)	69.51	< 0.001	1.00
≥ 55	476(55.67)	249(64.68)	60.74		1.52(1.23,1.87)
BMI (kg/m^2^)					
18.5–23.9	516(60.35)	231(60.00)	65.23	0.006	1.00
< 18.5	130(15.20)	83(21.56)	52.47		1.37(1.06,1.76)
≥ 24	209(24.45)	71(18.44)	71.34		0.83 (0.64,1.09)
Occupation					
farmer	301(35.20)	192(49.87)	61.16	0.014	1.00
worker	166(19.42)	85(22.08)	66.55		0.81(0.62,1.04)
office worker	388 (45.38)	108(28.05)	67.41		0.71 (0.56,0.90)
Origin					
rural area	476(55.67)	239(62.08)	61.90	0.028	1.00
urban area	371(43.39)	143(37.14)	67.42		0.77(0.62,0.94)
Education					
none	63(7.88)	20(5.54)	72.37	0.114	1.00
≤ 9 years	584(73.09)	302(83.66)	62.83		1.11(0.70,1.74)
> 9 years	152(19.03)	39(10.80)	71.32		0.73(0.42,1.26)
Family history					
no	831(97.19)	371(96.36)	64.09	0.882	1.00
yes	24(2.81)	14(3.64)	71.20		1.04 (0.61,1.78)
Tumor site					
tonge	333(39.08)	147(38.58)	62.63	0.001	1.00
gingiva	96(11.27)	52(13.65)	62.71		1.09 (0.78,1.51)
floor of the mouth	53(6.22)	40(10.50)	52.10		1.35(0.95,1.91)
cheek lining	110(12.91)	42(11.02)	69.54		0.85 (0.60,1.20)
palate	62(7.28)	33(8.66)	65.12		1.07(0.73,1.56)
lips	29(3.40)	9(2.36)	72.38		1.09(0.55,2.13)
oropharynx	27(3.17)	23(6.04)	43.91		2.08(1.34,3.23)
others	142(16.67)	35(9.19)	79.31		0.63(0.43,0.91)
Clinical stage					
I	136(18.89)	30(8.90)	79.75	< 0.001	1.00
II	220(30.56)	78(23.15)	69.95		1.66 (1.09,2.53)
III	109(15.14)	59(17.50)	57.56		2.70 (1.74,4.19)
IV	255(35.41)	170(50.45)	53.90		2.98(2.02,4.40)
Pathological grading					
well	336(49.48)	136(40.36)	67.63	0.001	1.00
moderate	272(40.06)	138(40.95)	63.01		1.28(1.01,1.62)
poor	71(10.46)	63(18.69)	51.66		1.64(1.21,2.20)
Surgery					
no	58(6.78)	86(22.34)	14.65	< 0.001	1.00
yes	797(93.22)	299(77.66)	70.12		0.18 (0.14,0.24)
Treatment					
surgery	316(39.65)	74(19.22)	72.59	0.211	1.00
surgery + CT	166(20.83)	70(18.18)	74.22		1.00 (0.72,1.40)
surgery + RT	111(13.93)	36(9.35)	72.75		0.97(0.65,1.44)
surgery + CRT	204(25.59)	119(30.91)	64.50		1.31 (0.98,1.76)
Smoking status					
no	596(69.71)	244(63.38)	65.97	< 0.001	1.00
yes	259(30.29)	141(36.62)	61.35		1.49(1.21,1.83)
Drinking status					
no	692(80.94)	285(74.03)	66.05	< 0.001	1.00
yes	163(19.06)	100(25.97)	58.35		1.52(1.21,1.91)

Abbreviations: CT, chemotherapy; RT, radiotherapy; CRT, chemoradiotherapy.

1240 oral cancer patients were followed up for 49235.00 person months and the overall survival rate for 1 year, 3 year and 5 year were 90.24%,75.26%, and 64.38% respectively.

### Univariate analysis of potential prognosis factors in oral cancer patients

An univariate Cox regression analysis was performed to test whether the sociodemographic and clinicopathological characteristic selected was associated with survival. As shown in Table 1, gender, age, BMI, occupation, origin, smoking, drinking, tumor site, clinical stage, histological grading, and surgery were all possible prognosis factors. Age ≥ 55 [1.52(1.23, 1.87)], BMI < 18.5 kg/m^2^ [1.37(1.06, 1.76)], smoking [1.49(1.21, 1.83)], drinking [1.52(1.21,1.91)], advanced clinical stage [1.66 (1.09, 2.53), 2.70 (1.74, 4.19), 2.98(2.02, 4.40) for stage II, III, IV respectively)] and poorly histological differentiation [1.64(1.21, 2.20)] were all indicators of poor survival, while female [0.67 (0.54, 0.84)], office worker [0.71 (0.56,0.90)], urban area [0.77(0.62, 0.94)], and surgery [0.18 (0.14, 0.24)] were protecting factors. Compared with tongue tumor, oropharynx tumor expects a poorer prognosis [2.08(1.34, 3.23)], while other tumor sites expects a better prognosis [0.63(0.43, 0.91)].

### Stratification analysis for evaluating the effect of treatment according to clinical stage and pathological grading

In the univariate Cox regression model, surgery is a potential protective factors. However neither chemotherapy nor radiotherapy was found significantly associated with the prognosis of oral cancer. Thus we made further analysis of the effects of treatment on survival in different clinical and pathological grading separately, results of which were presented in Table 2. For oral cancer patients with earlier clinical stage (I–II), when compared with surgery alone, surgery plus chemotherapy [0.91 (0.51, 1.63)], radiotherapy [0.92(0.41, 2.06)] or chemoradiotherapy [1.48 (0.84, 2.61)] did not shown significant statistic correlation with survival, after adjusting gender, age, BMI, occupation, origin, education, drinking, smoking, and family history. Similarly, in patients with advanced clinical stage (III-IV), when compared with surgery alone, surgery combined with chemotherapy [0.84 (0.53, 1.35)] or chemoradiotherapy [0.75(0.49, 1.13)] showed no better prognosis. Only surgery combined with radiotherapy [0.57(0.33, 0.99)] showed benefit effects. With regard to pathological grading, no significant differences were observed among surgery, chemotherapy, radiotherapy or chemoradiotherapy.

**Table 2:** Stratification analysis for evaluating the effect of treatment on survival [HR (95%CI)]

Treatment	Clinical stage^a^		Pathological grading^b^		
	I–II	III–IV	well	moderately	poorly
surgery	1.00	1.00	1.00	1.00	1.00
surgery + CT	0.91 (0.51,1.63)	0.84 (0.53,1.35)	0.72(0.39,0.43)	0.87(0.50,1.49)	1.70(0.59,4.89)
surgery + RT	0.92(0.41,2.06)	0.57(0.33,0.99)	0.88(0.43,1.81)	0.63(0.32,1.25)	0.833(0.23,3.03)
surgery + CRT	1.48 (0.84,2.61)	0.75(0.49,1.13)	1.14(0.67,1.95)	0.71(0.42,1.19)	1.51 (0.62,3.74)

^a^Adjusted for gender, age, BMI, occupation, origin, education, drinking, smoking, family history, pathological grading.

^b^Adjusted for gender, age, BMI, occupation, living area, education, drinking, smoking, family history, clinical stage

Abbreviation: CT, chemotherapy; RT, radiotherapy; CRT, chemoradiotherapy.

### Multivariate analysis of potential prognosis factors in oral cancer patients

All potentially significant factors derived from univariate model (*P* < 0.05) were incorporated into multivariate Cox proportional hazards logistic regression analyses using the stepwise backward method. As results shown in Table 3, patients with age ≥ 55 showed poorer overall survival [1.29(1.01, 1.61)] than age < 55, and BMI < 18.5 kg/m^2^ showed poorer overall survival [1.36(1.04, 1. 76)] than BMI of 18.5–23.9 kg/m^2^. Clinical stage II [1.71(1.09, 2.70)], III [2.50(1.54, 4.04)] and IV [2.46(1.58, 3.84)] were associated with poorer survival compared with stage I. Poor histological differentiation [1.46(1.04, 2. 06)] was also associated with worse survival. Patients with surgery exerted better survival [0.22(0.16, 0.31)] than patients without surgery, while patients from urban area had better survival [0.59(0.41, 0.83)] than rural area.

**Table 3:** Results of multivariate analysis of prognostic factors for survival in oral cancer

Variable	Overall survival		
	HR	95%CI	*P*
Age (≥ 55 vs < 55 )	1.29	1.01–1.61	0.041
Origin (Urban vs rural area )	0.59	0.41–0.83	0.003
Surgery (yes vs no)	0.22	0.16–0.31	< 0.001
Clinical stage			
I	1.00		
II	1.71	1.09–2.70	0.021
III	2.50	1.54–4.04	< 0.001
IV	2.46	1.58–3.84	< 0.001
Pathological grading			
well	1.00		
moderately	1.11	0.86–1.43	0.421
poor	1.46	1.04-2.06	0.029
BMI (kg/m^2^)			
18.5–23.9	1.00		
< 18.5	1.36	1.04-1.76	0.036
≥ 24	0.96	0.72–1.34	0.842

### Survival analysis

**Figure 1:**
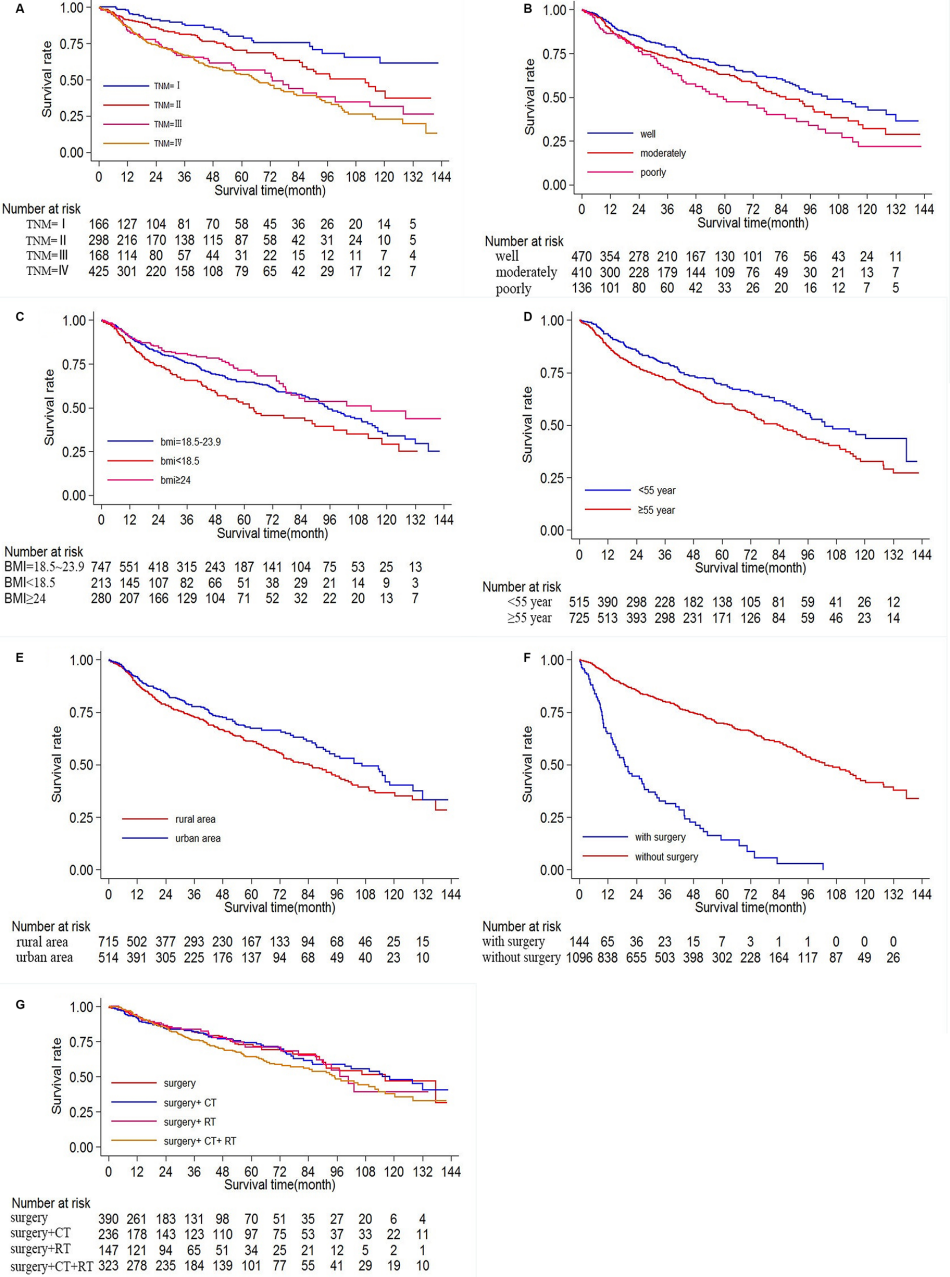
Ten-year survival of patients diagnosed with oral cancer in The First Affiliated Hospital of Fujian Medical University (Long-rank Mantel–Cox test) (**A**) Survival analysis according to clinical stage (stage I, II, III, IV), *P* < 0.001 by log rank test. (**B**) Survival analysis according to histological grade (poorly, moderately and well differentiated), *P* = 0.001. (**C**) Survival analysis according to BMI (< 18.5,18.5–23.9, ≥ 24 kg/m^2^), *P* = 0.006. (**D**) Survival analysis according to age(< 55, ≥ 55 years old), *P* < 0.001. (**E**) Survival analysis according to origin (rural area, urban area), *P* = 0.028. (**F**) Survival analysis according to surgery(with or without surgery), *P* < 0.001. (**G**) Survival analysis according to treatment(surgery alone, surgery +chemotherapy, surgery +radiotherapy, surgery + chemoradiotherapy, and *P* = 0.211.

Based on the Kaplan–Meier survival analysis, the overall 5-year survival rate was 64. 38%. The 5-year survival rates for stage I (*N* = 166), stage II (*N* = 298), stage III (*N* = 168) and stage IV (*N* = 425) were 79.75%, 69.95%, 57.56% and 53.90% respectively, with *P* < 0.001 by log rank test (Figure 1A), showing that the stage was a significant prognosis factor in the survival of oral cancer.

A significant difference existed in the overall 5-year survival rate depending on histopathological differentiation. The overall 5-year survival rate was 67.63% for the well differentiated type (*n* = 472), 63.01%for the moderately differentiated type (*n* = 410), and 51.66% for the poorly differentiated type (134), with *P* = 0.001 by log rank test (Figure 1B).

There was significant difference in the overall 5-year survival rate according to BMI, with 747 people belonging to BMI < 18.5 kg/m^2^, 213 people to BMI= 18.5–23.9 kg/ m^2^, 280 people to BMI ≥ 24 kg/m^2^. The overall 5-year survival rate decreased as BMI decreases, 77.68%, 69.29%, 61.64% for BMI ≥ 24 kg/m^2^, BMI= 18.5–23.9 kg/m^2^, BMI < 18.5 kg/m^2^, with *P* = 0.006 by log rank test (Figure 1C).

Significant differences were also found in the overall 5-year survival rate according to age (*P* < 0.001), origin (*P* = 0.028) and surgery *(P* < 0.001), results of which were shown in Figure 1D–1F.

However, no significant differences were found among different treatments. The overall 5-year survival rate was 72.59% for the surgery group (*n* = 390), 74.21% for the surgery+CT group (*n* = 236), 70.99% for the surgery + RT group (*n* = 147), 64.48% for the surgery +CRT group (*n* = 323), with *P* = 0.211 by log rank test result, which showed that no significant association between adjuvant treatment and survival rate (Figure 1G).

## DISCUSSION

Oral cancer is a disease strongly influenced by social factors [5, 7, 9]. Previous studies have demonstrated that the illness process is treated differently in the population with a lower level of education and income. This has led some authors to consider oral cancer as a disease that is characteristic of people with a low economic and educational level. In Brazil, patients with a lower income and education level had a higher mortality rate due to oral cancer [6]. Similar results were found in a study in Taiwan [9]. In this sample, univariate analysis found that office worker, origin of urban area were protective factors. In further analysis, patients of origin were of significance in the multiple Cox regression model, which indicating that patients with high economic levels have better prognosis and socioeconomic factors may have a strong influence on the delayed diagnosis and choice of treatment. However, we did not find education is related to survival in this population.

Several epidemiologic studies in different head and neck sites have examined the association between alcohol consumption and smoking prediagnosis and survival with conflicting results [10–13]. A multicenter population-based case–control study of laryngeal and hypopharyngeal cancer carried out in six European regions, reported that alcohol drinking affects the survival, albeit to a limited extent [14]. Stefania Boccia [15] stated that cigarette smoking was a negative prognostic factor for those current smokers before diagnosis of oral cavity cancer, and excessive alcohol use was associated with an increased hazard of overall survival. However, more recent studies that have examined the effect of alcohol drinking and smoking on survival reported opposite results [10, 11, 13]. In our study, univariate analysis showed that both drinking and smoking at diagnosis predict poor survival. However, these patterns did not persist after adjustment for significant prognostic factors in a multivariate model.

For a long time, obesity has been thought to be a preoperative risk factor for surgery due to its association with numerous complications, such as cardiovascular, pulmonary and metabolic disorders, which may result in increased postoperative morbidity [16]. However, previous authors have shown that obesity may be correlated with a favorable long-term prognosis in gastric cancer, head and neck cancer, breast cancer et al. [17–20]. Although a high BMI has been thought to be associated with a poorer short-term outcome than a low BMI due to simplicity and steadiness of surgical procedure in gastric cancer [21], the prognostic implications in oral cancer are unknown. In Our study, we showed that prognosis of the overweight patients was better than that of the normal- and low-BMI groups, even though the comparison between the normal- and high-BMI groups did not reach statistically significance. Multivariate analysis showed that a low BMI was an independent predictor of a poor prognosis. It is well known that patients with advanced cancer experience weight loss and poor nutrition, especially for oral cancer patients. Cancer lesions in the oral area may cause dysphagia, odynophagia, or alteration of taste and appetite, leading to a reduction of overall caloric intake and weight loss. Moreover, patients with adjuvant therapy are usually suffered from severe side effects, so low BMI patients are easily to be suffer from malnutrition, thus expecting worse prognosis. However, we did not find difference between normal- and high-BMI groups, probably because of the limited number of patients with extremely high BMI in a Chinese population. In fact, only 42(3.4%) patients are with a BMI ≥ 28 kg/m^2^ in the study, so the data may not be sufficient enough to reveal the association between obesity and prognosis.

For the treatment of oral cancer, it is still controversial. Usually, surgical treatment is preferred in the initial oral cancer, and the cases of progressed oral cancer with cervical lymph node metastasis can be provided with surgical treatment along with chemotherapy and radiation therapy [22]. Previous studies reported that overall survival and disease-specific survival was significantly higher in the surgically treated group compared with no surgery group in oral cavity squamous cell carcinoma [23–25]. Surgery and/or radiation therapy provides disease-specific survival benefit as compared with no therapy within the head and neck region [26]. On the other hand, some other studies indicated that radiotherapy plays an important role in the local control of small volume tumors, such as those of the T1–2 category [27–29], in head and neck squamous cell carcinoma. However, the efficacy of radiation therapy alone was shown to be reduced in cases of T3–4 category tumors. Addition of adjuvant radiotherapy to surgery did not significantly alter the 5-year local control rate or the overall survival rate in oral cavity squamous cell carcinoma patients with pT1–3N0 disease [30]. In our study we observed that patients underwent surgery had better survival than patients without, while combined with radiotherapy showed benefit effects in oral cancer patients of advanced clinical stage III-IV.

As for chemotherapy, it is usually applied to advanced stage, extracapsular spread, recurrence or metastasis. A main meta-analysis showed only a small significant survival benefit in favour of chemotherapy, so the routine use of chemotherapy is debatable [31]. Studies about oral cancer also showed that combination therapy with chemotherapy after surgical treatment is not significantly better than those who received only surgical treatment or surgery plus radiotherapy [23]. Results from this study suggest that surgery combined with chemotherapy or concurrent chemoradiotherapy may not be significantly associated with overall survival of oral cancer after adjusting for the effect of gender, age, BMI, occupation, origin, education, drinking, smoking, family history, clinical stage, pathological grading. One of the possible reasons for the controversial result about adjuvant therapy is that the use of adjuvant therapy is varied in different study population, for example, the agents, dose and cycles used for chemotherapy, as well as the dose and delivering for radiotherapy. Besides, sensitivity to adjuvant therapy may also differed from population to population. Another possible reason is that most patients with advanced oral cancer were administered with adjuvant therapy, data about patients without adjuvant therapy is limited, thus the potential benefit of adjuvant therapy may be unrevealed.

## MATERIALS AND METHODS

### Study subjects

Oral cancer patients were consecutively recruited from The First Affiliated Hospital of Fujian Medical University, within a period from January 2004 to December 2015. The inclusion criteria were as follows: 1) all cases were newly diagnosed primary oral cancer patients with histological confirmation; 2) all cases are Chinese Han population and reside in Fujian Province; 3) all cases were aged 20–80 years old. Exclusion criteria included recurrent oral cancer, metastasized cancer, previous chemotherapy or radiotherapy”. More than 90% oral cancer patients seen at Hospital consented to participate in our study. Initially 1377 patients were recruited, and 137 patients were exclude according to the inclusion/exclusion criteria”. So finally 1240 patients were included and were followed for up to 10 years, 110 patients were lost during the follow-up process.

The present study was approved by the Institutional Review Board (IRB) of Fujian Medical University, and has been performed in line with the ethical standards laid down in the 1964 Declaration of Helsinki and its later amendments. Informed consent was obtained from all individual participants included in the study.

### Methods

Sociodemographic information and lifestyle histories of patients were obtained at baseline by trained interviewers through the use of a standardized questionnaire. The subjects were considered smokers if they had smoked at least 100 cigarettes during their lifetime, and those who had consumed at least 1 drink/week for at least 6 months continuously were considered alcohol drinkers [32]. BMI values were categorized according to the Chinese definitions (< 18.5, 18.5–23.9, ≥ 24.0 kg/m^2^).

### Surgical treatment

Guidelines for surgery were in accordance with the recommendations of National Comprehensive Cancer Network, USA, Tumors were widely excised with > 1.5 cm safety margins (both peripheral and deep margins). Classic radical or modified neck dissections of levels I to V were performed in patients with clinically positive nodal disease. Supraomohyoid neck dissection of levels I to III was carried out in all patients with clinically negative necks in CT or MRI. Bilateral neck dissections were done when the primary tumor contacted or crossed the midline. Surgical defects were repaired with primary closure andlocal or vascularized free flaps.

### Postoperative radiotherapy/chemotherapy

Excluding those with poor general condition or unwilling to comply, Postoperative RT or concurrent chemoradiotherapy (CRT) was performed in patients with at least one of the following conditions: (1) a positive resection margin; (2) perineural invasion; (3) lymphovascular invasion; (4) ≥ 2 pathologically positive nodes; and (5) extracapsular extension of cervical nodes. RT or concurrent CRT was scheduled within 4 to 6 weeks after the operation.

As for radiation, the technique of 3D conformal RT or intensity modulated RT was used, with a linear accelerator using 6 to 10 MV X-ray to cover the surgical field with 1- to 2-cm margins and regional cervical lymphatics. For 3D conformal RT, the patients were initially treated with wedged-field technique to spare the contra lateral parotid and neck. The field covered the primary tumor and neck with a conventional fractionation of 2 Gy per fraction, 5 consecutive fractions per week for 44 to 50 Gy. Subsequently, the radiation field was narrowed down and focused on the primary tumor and metastatic neck toboost to a total dose of 60 to 66 Gy. For intensity modulated RT, dose painting with simultaneous integrated boost technique was applied. A total dose of 60 to 66 Gy were prescribed in 30 to 33 fractions with conventional fractionation. The beam angle was carefully selected to reduce the irradiation of the contralateral parotid and neck.

As for chemotherapy, oxaliplatin combined with paclitaxel or with 5-fluorouracil was used with the following dosage: oxaliplatin 130 mg/m^2^, paclitaxel 175 mg/m^2^, Q3W for 2 courses; oxaliplatin 130 mg/m^2^, 5-fluorouracil 450 mg/m^2^ Q2W for 3–6 courses.

### Follow-up

Follow-up data were obtained by phone, outpatient visits and our clinical database. Follow-up of all patients was carried out according to our standard protocol (every six months for at least 2 years, every twelve months for the next 3 years, and after 5 years every 24 months for life). The median follow-up time was 72 months for all patients. Overall survival rate was calculated from the date of diagnosis to the date of death from any cause or the date of the last follow-up observation. Data were censored at the date of death from causes not related to oral cancer or at the date of last follow-up.

### Statistical assessment

Potential prognostic factors included in the study was firstly analyzed by univariate Cox regression and then further tested by multivariate cox regression analysis with hazard ratio (HR) and 95% confidence intervals (CIs) calculated. Stratified analyses by potential effect modifiers were performed with adjusted Cox regression model. The survival rate was calculated using the Kaplan Meier method, and the log rank test was performed for significance test of the predicted prognosis factors. All analysis were performed using Statistical significance was considered at *P* < 0.05.
